# Supplementary material for: Exploration of O-GlcNAc transferase glycosylation sites reveals a target sequence compositional bias
Source: J Biol Chem. 2023 Mar 22;299(5):104629. doi: 10.1016/j.jbc.2023.104629 (PMC10164912; doi:10.1016/j.jbc.2023.104629)
Supplement: Supplemental Figures S1–S3, Tables S1 and S2 [file mmc1.docx]

**Supplementary Information for:**

**Exploration of O-GlcNAc transferase glycosylation sites reveals a target sequence compositional bias**

**Authors:** P. Andrew Chong, Michael L. Nosella, Manasvi Vanama, Roxana Ruiz-Arduengo, Julie D. Forman-Kay

Table S1. Expected and observed masses for various glycosylation states in Figure 1.

| Glycosylation state | Figure 1a. EWS | |
| --- | --- | --- |
|  | Expected | Observed |
| 0 | 27992.3 | 27992.3 |
| +4 | 28805.5 | 28804.8 |
| +5 | 29008.8 | 29008.0 |
| +6 | 29212.1 | 29211.0 |
| +7 | 29415.4 | 29414.5 |
| +8 | 29618.7 | 29617.8 |
| +9 | 29822.0 | 29821.1 |
| +10 | 30025.3 | 30024.8 |
|  | Figure 1b. FUS | |
| 0 | 21554.4 | 21554.5 |
| +1 | 21757.8 | 21758.0 |
| +2 | 21961.1 | 21961.0 |
| +3 | 22164.4 | 22164.2 |
| +4 | 22367.7 | 22367.9 |
|  | Figure 1c. TAF15 | |
| 0 | 22716.7 | 22717.7 |
| +1 | 22920.0 | 22921.0 |

Table S2. Sequences of WT and mutant FUS LCR_N_. Mutated positions are highlighted in red.

| Protein | Sequence |
| --- | --- |
| WT | MASNDYTQQA TQSYGAYPTQ PGQGYSQQSS QPYGQQSYSG YSQSTDTSGY GQSSYSSYGQ  SQNTGYGTQS TPQGYGSTGG YGSSQSSQSS YGQQSSYPGY GQQPAPSSTS GSYGSSSQSS  SYGQPQSGSY SQQPSYGGQQ QSYGQQQSYN PPQGYGQQNQ YNSSSGGGGG GGGGGNYGQD  QSSMSSGGGS GGGYGNQDQS GGGGSGGYGQ QDRG |
| Mut-A | MASNDYTQQA TQSYGAYPTQ PGQGYSQQSS **T**PY**A**Q**A**SYS**T** YS**P**ST**T**TS**A**Y GQSSYSSYGQ  SQNTGYGTQS TPQGYGSTGG YGSSQSSQSS YGQQSSYPGY GQQPAPSSTS GSYGSSSQSS  SYGQPQSGSY SQQPSYGGQQ QSYGQQQSYN PPQGYGQQNQ YNSSSGGGGG GGGGGNYGQD  QSSMSSGGGS GGGYGNQDQS GGGGSGGYGQ QDRG |
| Mut-B | MASNDYTQQA TQSYGAYPTQ PGQGYSQQSS QPYGQQSYSG YSQSTDTSGY GQSSYSSYGQ  SQNTGY**A**T**T**S TPQ**A**Y**P**ST**AP** Y**AT**S**P**SSQSS YGQQSSYPGY GQQPAPSSTS GSYGSSSQSS  SYGQPQSGSY SQQPSYGGQQ QSYGQQQSYN PPQGYGQQNQ YNSSSGGGGG GGGGGNYGQD  QSSMSSGGGS GGGYGNQDQS GGGGSGGYGQ QDRG |
| Mut-C | MASNDYTQQA TQSYGAYPTQ PGQGYSQQSS QPYGQQSYSG YSQSTDTSGY GQSSYSSYGQ  SQNTGYGTQS TPQGYGSTGG YGSSQSSQSS YGQQSSYP**A**Y **T**QQPAP**TT**TS **A**SY**AT**SSQSS  SYGQPQSGSY SQQPSYGGQQ QSYGQQQSYN PPQGYGQQNQ YNSSSGGGGG GGGGGNYGQD  QSSMSSGGGS GGGYGNQDQS GGGGSGGYGQ QDRG |
| Mut-D | MASNDYTQQA TQSYGAYPTQ PGQGYSQQSS **T**PY**A**Q**A**SYS**T** YS**P**ST**T**TS**A**Y GQSSYSSYGQ  SQNTGY**A**T**T**S TPQ**A**Y**P**ST**AP** Y**AT**S**P**SSQSS YGQQSSYPGY GQQPAPSSTS GSYGSSSQSS  SYGQPQSGSY SQQPSYGGQQ QSYGQQQSYN PPQGYGQQNQ YNSSSGGGGG GGGGGNYGQD  QSSMSSGGGS GGGYGNQDQS GGGGSGGYGQ QDRG |
| Mut-E | MASNDYTQQA TQSYGAYPTQ PGQGYSQQSS QPYGQQSYSG YSQSTDTSGY GQSSYSSYGQ  SQNTGY**A**T**T**S TPQ**A**Y**P**ST**AP** Y**AT**S**P**SSQSS YGQQSSYP**A**Y **T**QQPAP**TT**TS **A**SY**AT**SSQSS  SYGQPQSGSY SQQPSYGGQQ QSYGQQQSYN PPQGYGQQNQ YNSSSGGGGG GGGGGNYGQD  QSSMSSGGGS GGGYGNQDQS GGGGSGGYGQ QDRG |
| Mut-F | MASNDYTQQA TQSYGAYPTQ PGQGYSQQSS **T**PY**A**Q**A**SYS**T** YS**P**ST**T**TS**A**Y GQSSYSSYGQ  SQNTGY**A**T**T**S TPQ**A**Y**P**ST**AP** Y**AT**S**P**SSQSS YGQQSSYP**A**Y **T**QQPAP**TT**TS **A**SY**AT**SSQSS  SYGQPQSGSY SQQPSYGGQQ QSYGQQQSYN PPQGYGQQNQ YNSSSGGGGG GGGGGNYGQD  QSSMSSGGGS GGGYGNQDQS GGGGSGGYGQ QDRG |


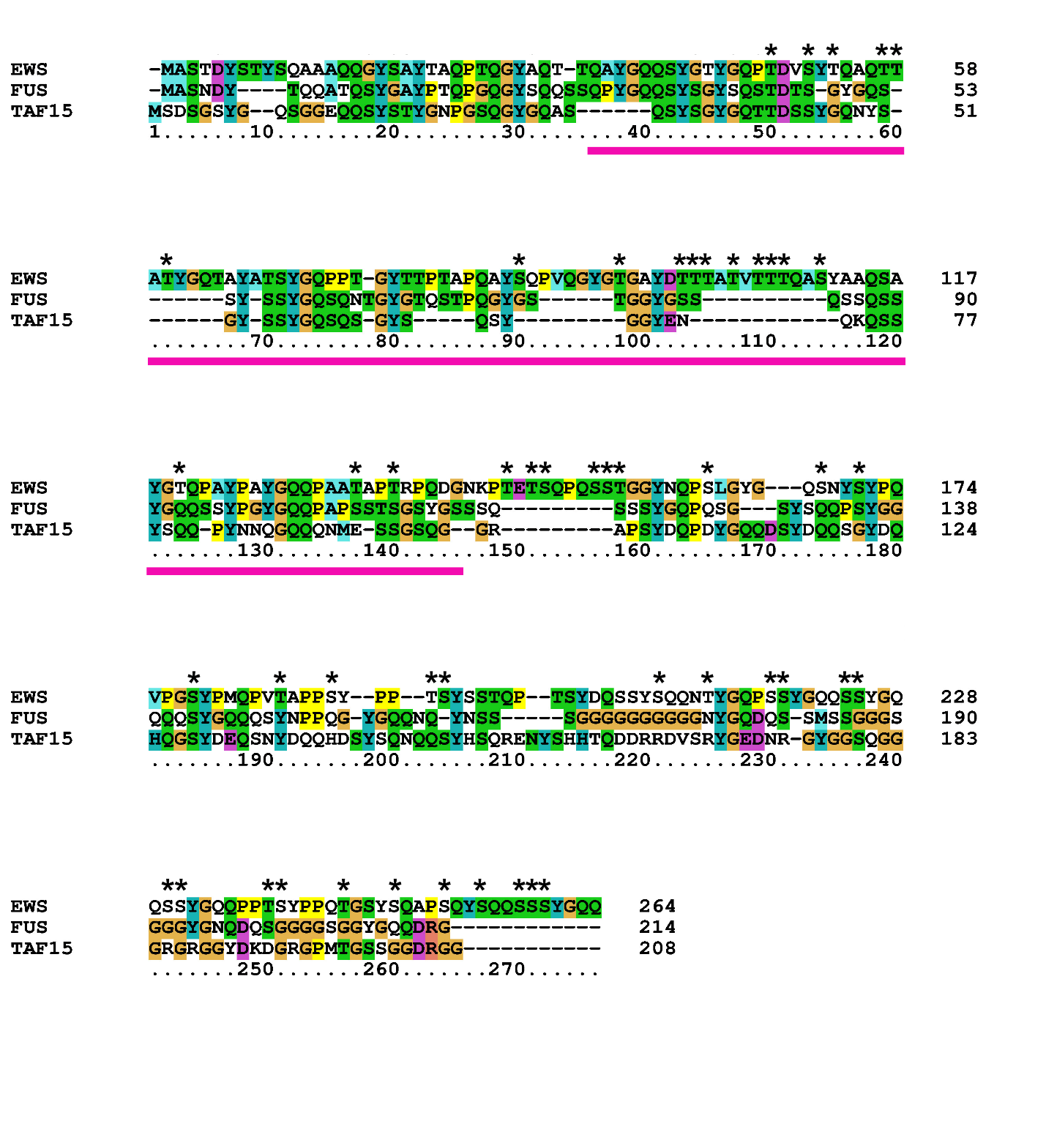
 Figure S1: Alignment of the LCR_N_ regions of the FET proteins. Possible glycosylation sites in EWS are highlighted with asterisks. The region mutated in the FUS compositional mutants is highlighted by a violet colored bar.


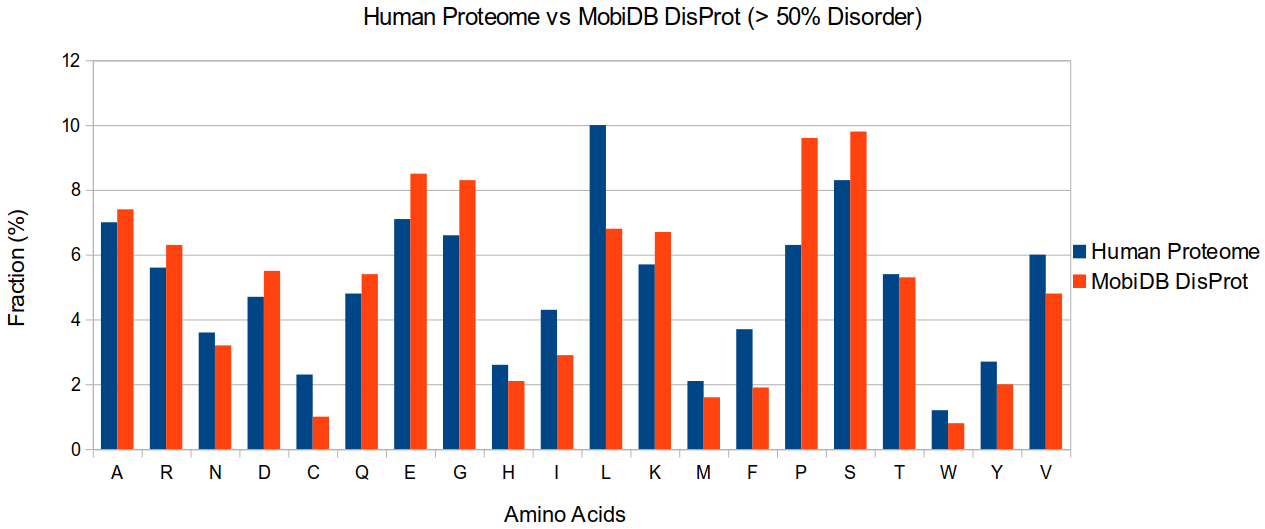


Figure S2: Amino acid proportions in the human proteome and a set of disordered proteins. For the human proteome, we used all human proteins in the Uniprot database. For the disordered set, we used a manually curated MobiDB version of the DisProt database and selected for human proteins with greater than 50% defined fractional disorder content.


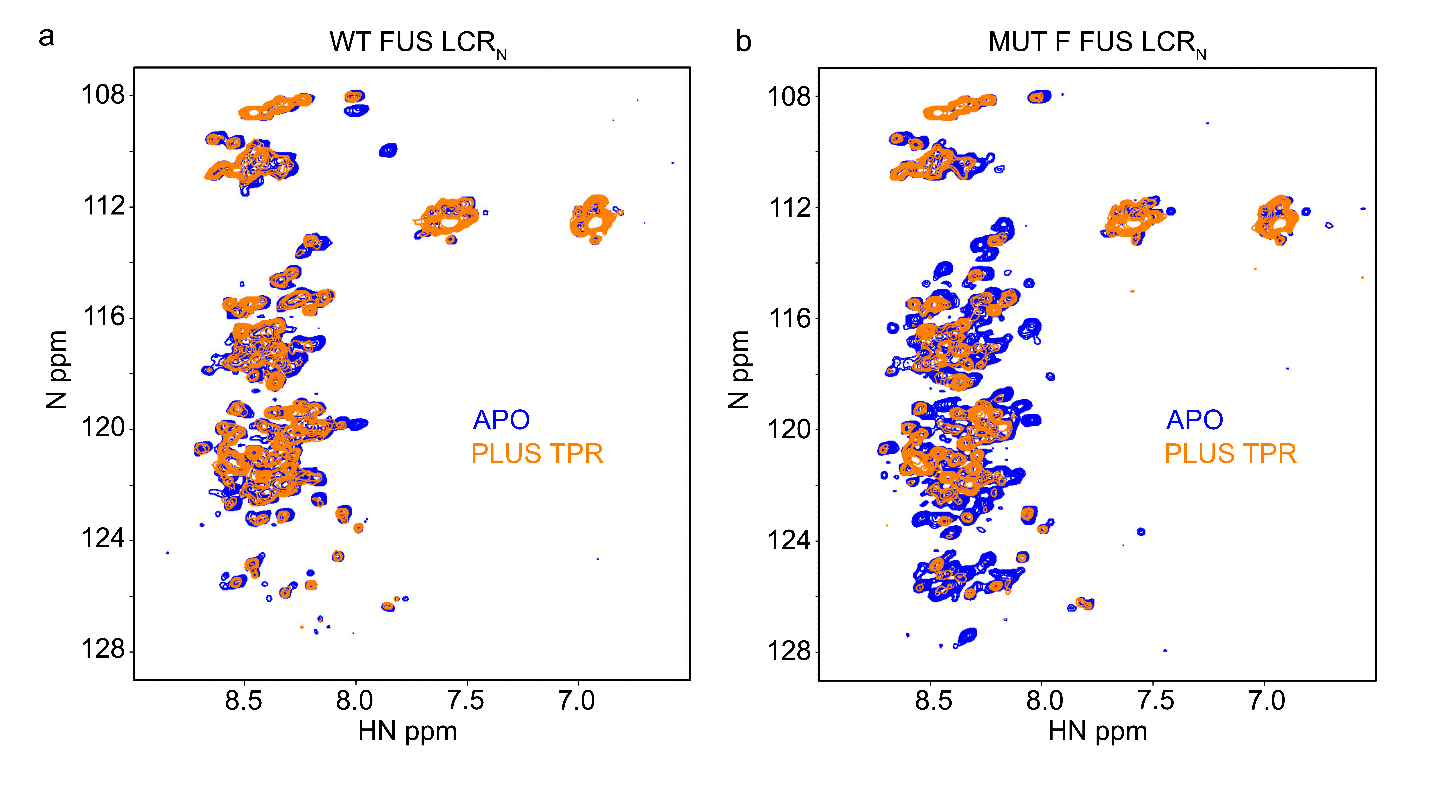
Figure S3: ^1^H-^15^N HSQC spectra of WT FUS LCR_N_ (a) and Mut-F FUS LCR_N_ (b) in the presence and absence of OGT-TPR. Spectra of the FUS LCR_N_ at 20 µM are shown in blue and spectra in the presence of 50 µM OGT-TPR are shown in orange. Spectra were recorded with a field strength of 600 MHz at 5°C in a buffer comprised of 40 mM KPO_4_, 125 mM NaCl, 0.5 mM EDTA, 0.5 mM benzamidine, 5 mM DTT and 10% D_2_O, pH 7.2.
